# Generalized 3D registration algorithm for enhancing retinal optical coherence tomography images

**DOI:** 10.1117/1.JBO.29.6.066002

**Published:** 2024-05-14

**Authors:** Tiffany Tse, Yudan Chen, Mahsa Siadati, Yusi Miao, Jun Song, Da Ma, Zaid Mammo, Myeong Jin Ju

**Affiliations:** a The University of British Columbia, School of Biomedical Engineering, Faculty of Medicine and Applied Science, Vancouver, British Columbia, Canada; b The University of British Columbia, Department of Ophthalmology and Visual Sciences, Faculty of Medicine, Vancouver, British Columbia, Canada; c Wake Forest University, School of Medicine, Winston-Salem, North Carolina, United States

**Keywords:** optical coherence tomography, angiography, ophthalmology, image registration, image processing, retinal imaging

## Abstract

**Significance:**

Optical coherence tomography (OCT) has emerged as the standard of care for diagnosing and monitoring the treatment of various ocular disorders due to its noninvasive nature and *in vivo* volumetric acquisition capability. Despite its widespread applications in ophthalmology, motion artifacts remain a challenge in OCT imaging, adversely impacting image quality. While several multivolume registration algorithms have been developed to address this issue, they are often designed to cater to one specific OCT system or acquisition protocol.

**Aim:**

We aim to generate an OCT volume free of motion artifacts using a system-agnostic registration algorithm that is independent of system specifications or protocol.

**Approach:**

We developed a B-scan registration algorithm that removes motion and corrects for both translational eye movements and rotational angle differences between volumes. Tests were carried out on various datasets obtained from two different types of custom-built OCT systems and one commercially available system to determine the reliability of the proposed algorithm. Additionally, different system specifications were used, with variations in axial resolution, lateral resolution, signal-to-noise ratio, and real-time motion tracking. The accuracy of this method has further been evaluated through mean squared error (MSE) and multiscale structural similarity index measure (MS-SSIM).

**Results:**

The results demonstrate improvements in the overall contrast of the images, facilitating detailed visualization of retinal vasculatures in both superficial and deep vasculature plexus. Finer features of the inner and outer retina, such as photoreceptors and other pathology-specific features, are discernible after multivolume registration and averaging. Quantitative analyses affirm that increasing the number of averaged registered volumes will decrease MSE and increase MS-SSIM as compared to the reference volume.

**Conclusions:**

The multivolume registered data obtained from this algorithm offers significantly improved visualization of the retinal microvascular network as well as retinal morphological features. Furthermore, we have validated that the versatility of our methodology extends beyond specific OCT modalities, thereby enhancing the clinical utility of OCT for the diagnosis and monitoring of ocular pathologies.

## 1 Introduction

Optical coherence tomography (OCT) is a high-resolution, noninvasive, three-dimensional (3D), *in vivo* imaging technique that enables cross-sectional imaging of a sample at a micrometer resolution.^1^^,^^2^ Among other imaging technologies, OCT is a relatively new imaging technique with widespread applications in medicine, especially in ophthalmology.^3^^–^^5^ As OCT is becoming a standard of care for diagnosing and monitoring the treatment of various ocular diseases, its current applications in ophthalmology involve the study of retinal diseases, such as glaucoma,^6^ retinal detachments,^7^ and age-related macular degeneration,^8^^,^^9^ all of which can cause permanent vision loss if left untreated. Additionally, OCT-based angiography (OCTA) is a functional extension of OCT wherein repeated scans are acquired at the same transverse location, and their variation is subsequently measured to distinguish a flow signal from a static sample.^10^^–^^12^ This enables *in vivo* visualization of tissue structure and blood flow through retinal vessels and choroidal capillaries without the use of contrast agents.

In order to navigate the imaging location and mitigate motion artifacts, patients are required to visually fixate on a target during the OCT image acquisition. However, conventional OCT methods are still susceptible to motion artifacts due to involuntary eye movements from respiratory and cardiac dynamics as well as microsaccades and blinking, which can adversely affect the image quality. Furthermore, poor eye fixation commonly occurs in patients with impaired focal vision, resulting in more severe motion artifacts and at a higher frequency compared to healthy individuals.^13^^,^^14^ Both hardware-based and software-based strategies have been previously proposed to mitigate these motion artifacts.^15^^,^^16^ Hardware-based solutions employ either motion-tracking systems mounted on the OCT machine to rescan the motion-detected B-scans in real time,^17^^–^^20^ or high-speed imaging modalities that can acquire images faster than eye movement.^21^^–^^23^ However, implementing motion-tracking hardware and increasing the acquisition speed typically leads to an increase in system complexity and cost, while still being unable to fully address various motion artifacts. For instance, motion-tracking systems are unable to compensate for eye motion with large amplitudes, and as such, capillary distortions and B-scan rotations will often appear in OCT images as discontinuous vessel segments.^24^^,^^25^ Previous studies have demonstrated the efficacy of high-speed imaging systems in reducing motion artifacts; however, a discernible compromise exists between acquisition speed and signal-to-noise ratio (SNR), consequently yielding a diminished quality in the acquired data.^26^

Alternatively, software-based approaches correct motion artifacts by acquiring and averaging multiple volumes.^27^^–^^30^ These methods require volume registration to spatially align the repeated volumes prior to averaging, in order to correct subtle variations between volumes caused by the involuntary movement from the patient during image acquisition. Available OCT registration algorithms can be categorized into image feature-based methods and volumetric transformation-based methods.^31^ Feature-based methods identify distinct anatomical features to determine transformation parameters. A two-dimensional (2D) motion-correction approach has been recently explored, based on microsaccade-free *en face* strips, which are derived from OCTA volumes and registered using scale-invariant feature transform keypoints.^32^ Although this method was able to produce exquisite 2D images from diabetic retinopathy (DR) patients, most of the depth information in the volumes was discarded, which could have been useful data for extending the registration to 3D. Additionally, our recent work demonstrated an *en face* feature-based study to create a template-less, 3D registration, and averaging methodology by automatically stitching small, motion-free subvolumes.^28^ However, despite its ability to produce advanced 3D renderings demonstrated on DR patients, this method may be less effective in cases where there are inadequate *en face* features, or in cases with severe microsaccades and axial motion.

On the other hand, volumetric transformation-based methods aim to maximize the similarities between the reference and the target volumes.^31^ A recent 3D OCT/OCTA registration method has been demonstrated to be robust in suppressing motion artifacts and improving SNR, but may fail if significant intervolume motion is present.^27^

Another software-based approach for the registration of high-resolution adaptive-optics OCT (AO-OCT) retinal images adopts coarse-to-fine B-scan registration to correct translational eye movements based on a single reference volume, followed by A-line registration to a global coordinate system by repeating the B-scan registration with multiple reference volumes using 3D phase-only correlation (POC) and normalized cross correlation (NCC).^29^ Although this method can produce outstanding results from a high-resolution cone mosaic layer, the global A-line registration is less effective on retinal layers with damaged structure and images with lower resolution than AO-OCT.

In this study, we iterate upon our previous works^28^^,^^32^ and the algorithm introduced by Kurokawa et al.^29^ to introduce a robust and software-based 3D registration algorithm. The proposed method applies both B-scan and affine registrations to correct intervolume translational shifts and rotational angle differences, respectively. Our method is distinct from previous algorithms in this domain which have typically been tailored toward particular OCT specifications or concentrate only on one single pathology. The work presented is effective in detecting and correcting for motion artifacts across diverse OCT modalities including OCTA, encompassing differences in field-of-view (FOV), axial and lateral resolution, and SNR. As a result, our approach enables us to obtain high-quality volumes from motion-corrupted data acquired by various OCT systems, thereby expanding our algorithm to many applications and ultimately improving the clinical utility of OCT technology. Here we present our validation of the algorithm performance on three different systems, using two different metrics for quantitative evaluation to demonstrate its versatility through successful application to a diverse array of datasets. Examples of potential applications include multivolume averaging,^33^ longitudinal clinical studies using molecular contrast imaging,^34^ and dynamic imaging by evaluating spatial and temporal differences between consecutive volumes.^35^^,^^36^ These applications demonstrate the versatility and potential impact of our algorithm across a range of clinical and research contexts.

## 2 Methods

Retinal images were acquired by two custom-built OCT systems with different system characteristics and a commercially available swept-source OCT (SS-OCT) system, including a custom-built dual-spectrometer OCT with high-SNR and high acquisition speed mode (DS-OCT),^37^^,^^38^ a high lateral resolution sensorless adaptive optics OCT (SAO-OCT),^39^^,^^40^ and the PLEX Elite 9000 Swept-Source OCT (Carl Zeiss Meditec, Dublin, California, USA). Table 1 summarizes the specifications of the systems used for this study. The custom-built OCT systems did not have hardware-based motion-tracking capabilities; instead, a visual target was used for fixation during image acquisition.

**Table 1:** Summary of system specifications.

System	Wavelength (nm)	Field of view (mm×mm)	Axial resolution (μm)	Lateral resolution (μm)
DS-OCT	810	6 × 6	3.2	9.7
SAO-OCT	1060	0.5 ×0.5	7.0	3.3
PLEX Elite 9000	1060	6 × 6	6.3	20

The subjects were recruited and imaged at the Eye Care Centre in Vancouver General Hospital (Vancouver, British Columbia, Canada) in accordance with the tenets of the Declaration of Helsinki. Each subject was informed of the nature of the study and its implications and asked to sign a consent form before any study procedures or examinations were conducted.

### 2.1 Preprocessing

**Fig. 1:**
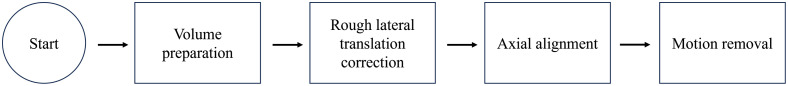
Illustration of preprocessing steps of multivolume registration and averaging algorithm.

There are four steps in the processing pipeline, including volume preparation, rough lateral translation correction, axial alignment, and motion removal, all of which were performed prior to the multivolume registration and averaging process as demonstrated in Fig. 1. Each of the processing steps will be described in detail throughout Secs. 2.1.1–2.1.4.

#### 2.1.1 Volume preparation

The acquired raw OCT data were processed using the traditional OCT processing methods, such as wave number resampling,^41^ numerical dispersion compensation,^42^ fast Fourier transform, and axial motion correction. Motion correction for each volume was performed in both fast and slow B-scan directions, by selecting the center frame as the reference. Each frame within the volume is shifted axially to achieve maximum cross correlation with respect to the reference frame. After motion correction has been performed on all acquired volumes, the volumes are ranked from highest to lowest quality based on a quantitative metric, which considers the level of motion, SNR, and image clarity. The volume with the highest ranking is selected as the reference for the remainder of the registration procedure.^29^

#### 2.1.2 Transverse alignment

An approximate translational shift between the selected target and the reference volumes was estimated using the 2D POC.^43^ We generated the mean projection of the reference and target volumes and computed the 2D Fourier transform of the *en face* images as shown in Eq. (1), denoted by $G_{r}$ and $G_{t}$, respectively. The normalized cross-power spectrum was calculated using

$$R=\frac{G_{r}\circ G_{t}^{*}}{\left|G_{r}\circ G_{t}^{*}\right|},$$ (1)

where ∘ and * denote the element-wise multiplication and complete conjugate operation, respectively. The translational shifts of the target image (Δx,Δy) relative to the reference image are estimated using the maximum value of the inverse Fourier transform of the normalized cross-power spectrum $r=F^{-1}(R)$:

$$(\Delta x,\Delta y)=\underset{\left(x,y\right)}{\arg\;\max}\;\{r\}.$$ (2)

The 2D POC can also be extended to 3D to find the rotational and scaling differences by performing 2D POC layer-by-layer, traversing in depth of the volume. However, only translational shifts between the two *en face* projection images were considered in this step for initial transverse alignment.

In the commercial OCT system, the motion-tracking setting uses active feedback control to reduce artifacts caused by eye movements, such as blinking and saccades during scanning.^16^ For OCT data acquired with motion-tracking on, an additional nonrigid alignment step was added to preprocessing (Sec. 2.1)^42^^,^^43^ to compensate for the randomized coordinates and different scanning orientations caused by rescanning the location of motion. This nonrigid registration aligns the reference and target volumes using the *en face* projections extracted from the machine. The transformation matrix obtained from the 2D projection is then applied to all the layers of the target volume. Since this method is feature-based, more features in the image will result in better registration.

#### 2.1.3 Axial alignment

**Fig. 2:**
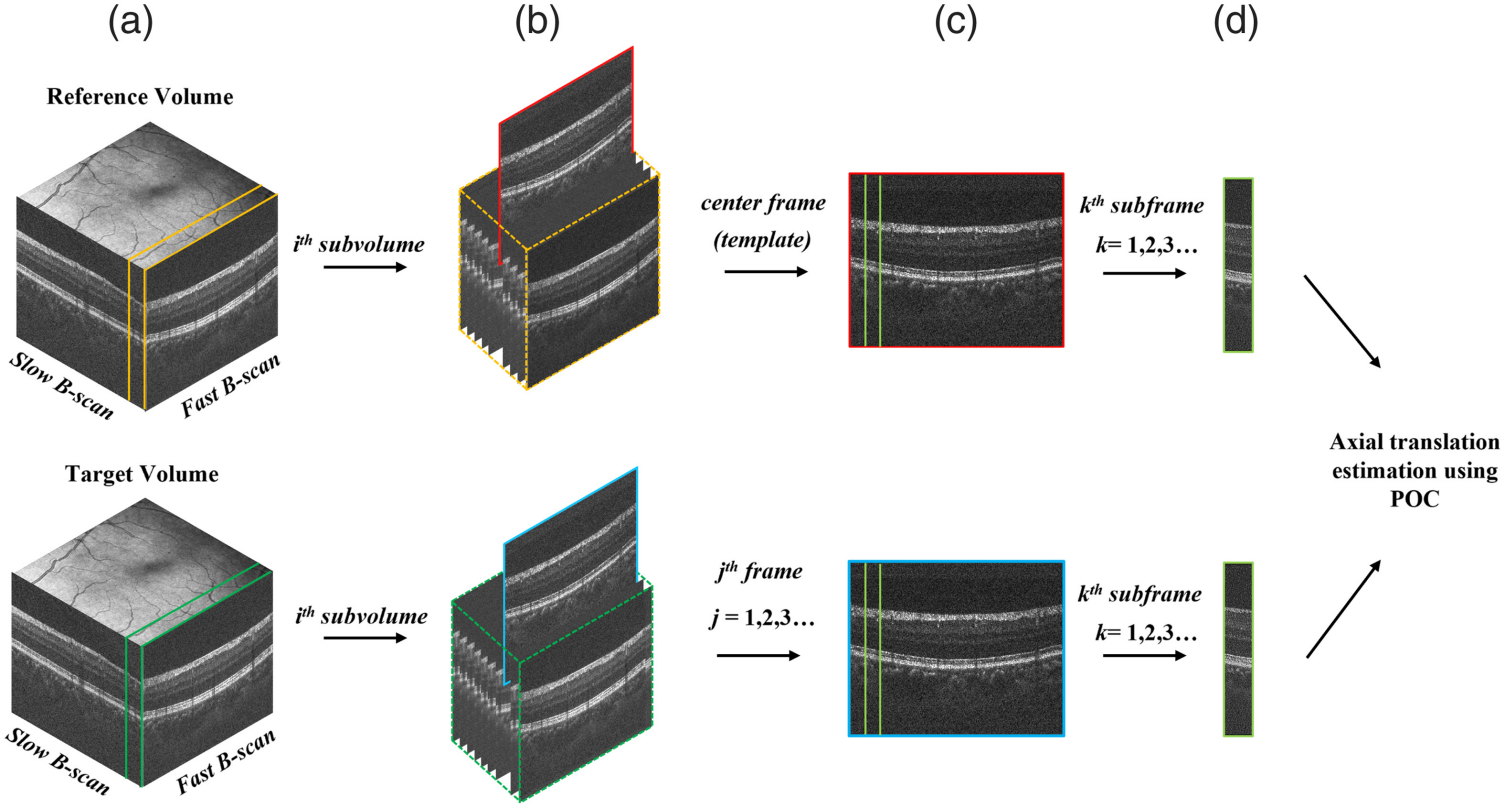
Illustration of the axial alignment process. (a) Reference and target volumes are divided into subvolumes. (b) The center frame of the reference subvolume is used to align all frames in the target subvolume. (c) The center frame is divided into subframes to align all subframes in the target frame. (d) The axial shift is estimated and applied to each subframe.

Following the 2D translation correction and nonrigid alignment of the volumes, axial alignment is required to expand the registration up to 3D. Figure 2 illustrates a schematic representation of this algorithm. First, the reference and target volumes were divided into subvolumes with a fixed number of fast B-scans. Each reference subvolume requires a reference frame, which we have chosen as the center B-scan, to align all B-scans in the target subvolume. For example, if each subvolume consisted of 10 fast B-scans, the reference frame would be selected as the fifth B-scan. The reference frame and each target B-scan were then partitioned into sub-B-scans with the width set to a fixed number of A-lines. The 2D POC [Eq. (1)] was applied to each reference sub-B-scan to calculate the axial shift, which was applied to the target sub-B-scans.

#### 2.1.4 Motion removal

**Fig. 3:**
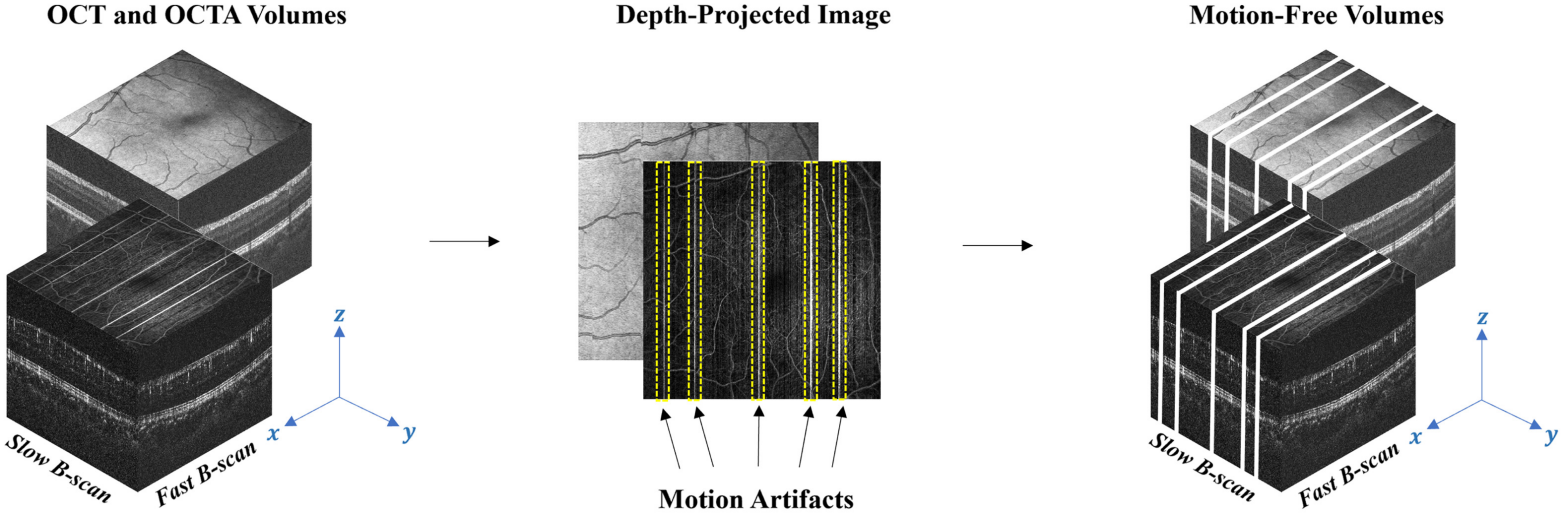
Illustration of automatic motion detection and removal process. Motion artifacts are enclosed by yellow dashed lines.

Each OCT volume has a unique pattern of distortion along the slow B-scan direction due to motion artifacts. Microsaccades and small capillary distortions can manifest as abrupt shifts OCT images, which appear as blood vessel displacement or discontinuity. In order to automatically detect and remove these artifacts, the cross correlation of each pair of adjacent fast B-scans was calculated in a bidirectional manner, beginning in the center frame and progressing outward toward the ends of the volume. A similar algorithm was introduced in our previous work,^28^ which enabled the detection of motion artifacts from OCTA *en face* images. However, we have developed an alternate algorithm capable of detecting motion directly from OCT volumes, since OCTA is not available in all OCT systems. In this method, each pair of adjacent B-scans was analyzed in terms of its offset (Δx) (translation along the fast scan direction) from its corresponding location of maximum cross correlation. Subsequently, the pixel locations containing these offsets, along with three additional pixels on either side, were removed for the complete removal of motion artifacts as illustrated in Fig. 3.

### 2.2 3D Registration

**Fig. 4:**
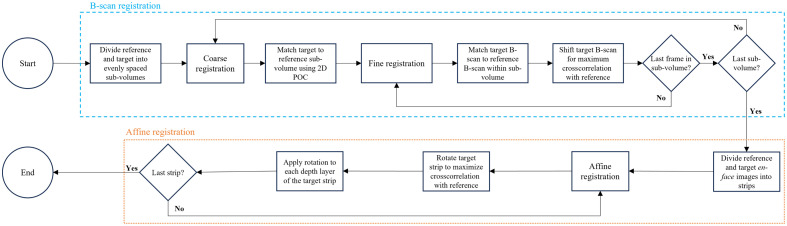
Illustration of 3D registration steps, including coarse-to-fine and affine procedures.

There are two major components in the 3D registration. This iterative process is illustrated in Fig. 4, where the blue dashed lines represent the coarse-to-fine B-scan registration and the orange dotted lines represent the affine registration. Each of the these steps will be described in detail in Secs. 2.2.1 and 2.2.2.

#### 2.2.1 B-scan registration

Similar to the B-scan registration method presented by Kurokawa et al.,^29^ the coarse-to-fine approach was employed to register multiple target volumes to a single reference volume. In our registration process, each target volume was divided into evenly spaced subvolumes consisting of a number of sequential fast B-scans, which should be sufficiently small to avoid any motion within the subvolume. Using the 2D POC described in Eq. (1), we computed the relative displacement of each target subvolume (Δx,Δy) to the reference volume to match the target subvolume to the most suitable reference subvolume. As described in Sec. 2.1.3, all the volumes have already been aligned axially, rendering the depth displacement Δz negligible during this stage. For the fast B-scan locations that were not matched, linear interpolation was computed to estimate the displacement.

Following the coarse registration of subvolumes, we computed the displacement of each target fast B-scan with respect to its best-matching reference frame at pixel-level precision. This fine registration step is distinct from the preceding coarse registration by refining the search space to the reference subvolume, rather than encompassing the entire volume. Additionally, we compared the shift amount of each fast B-scan with its adjacent B-scans to correct errors in the coarse-to-fine procedure. If the difference in the shift amount exceeds a predefined tolerance value, which we have empirically determined to be between 3 and 5 pixels, it was presumed to be an improper match and the B-scan was excluded. Interpolation and extrapolation were subsequently applied to reconstruct the removed B-scans due to error, and the registered volumes were constructed by shifting each B-scan based on the calculated Δx (translation along the fast scan direction) and Δy (translation along the slow scan direction).

#### 2.2.2 Affine registration

**Fig. 5:**
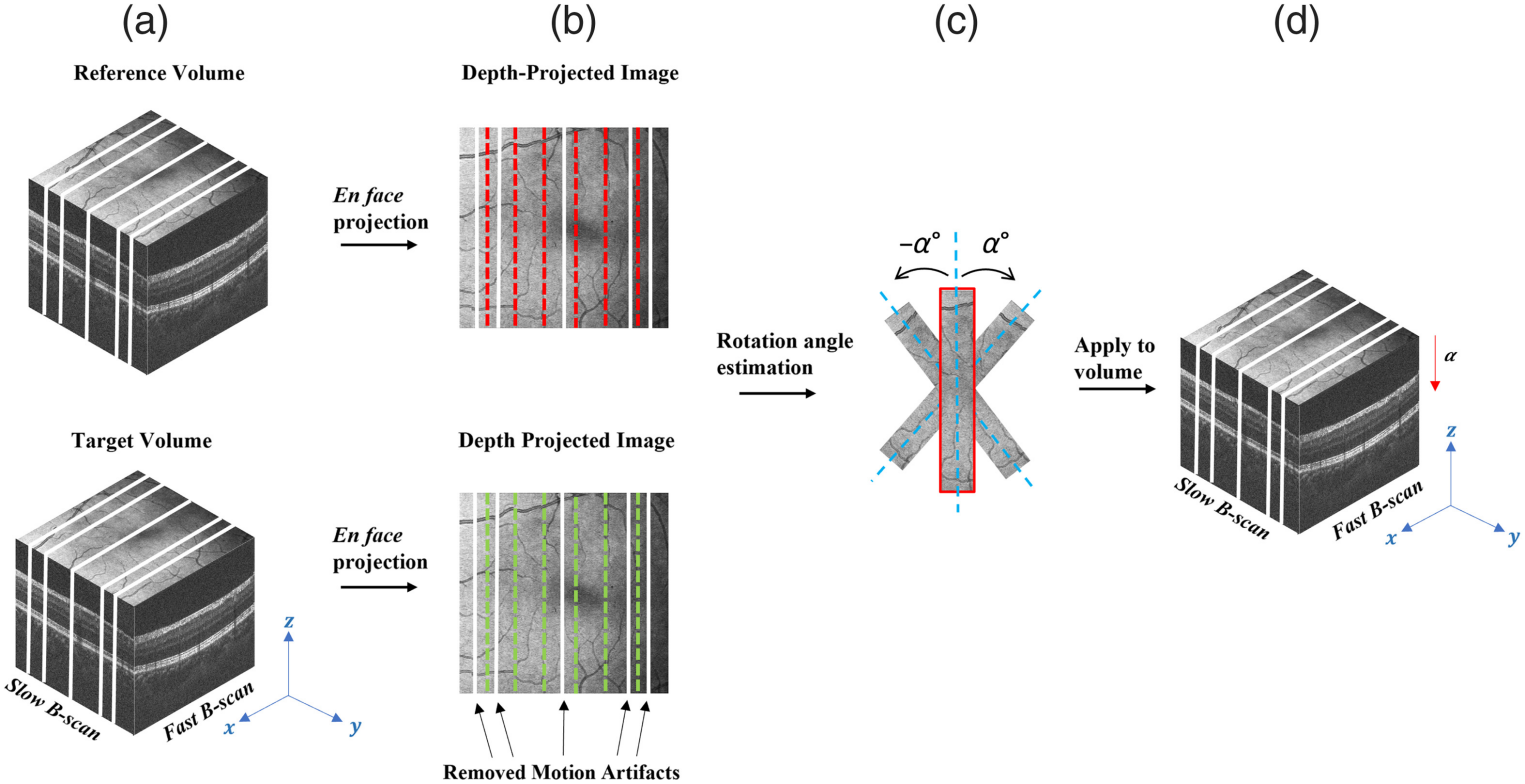
Illustration of affine registration process. (a) Depth projection of the reference and target volumes. (b) The *en face* images are divided into evenly spaced strips. (c) Rotation angle estimation is performed, where α is between ±1  deg. (d) The rotational angle of each strip is applied to all depth positions of the target volume.

Affine registration compensates for the angle discrepancy between the reference and target volumes by rotating each target frame to achieve the maximum cross-correlation relative to its corresponding reference frame. After the coarse-to-fine procedure, the partially registered target and reference volumes were projected along the depth to generate the *en face* images. Reference and target *en face* images were divided into the same number of equally spaced strips as shown by the dotted lines in Fig. 5(b). The affine registration was performed by rotating each target strip using a binary search approach until the cross correlation between the reference and target strips reached a maximum value. Finally, the rotation angle obtained from the *en face* projections was applied to all depth indices of the strip to correct each target subvolume. Since the rotation angle is often very small, it could be assumed that it is within the range of ±1  deg. This comprehensive approach ensures that rotational differences throughout the volume are effectively corrected, providing enhanced alignment between the reference and target volumes.

### 2.3 Quantitative Evaluation Metrics

Currently, there is no gold standard method for evaluating the performance of image registration algorithms, and measuring the accuracy of the registration remains a challenge. However, there are several reliable quantitative methods that may be used to approximate registration performance. In this study, the image quality improvement was evaluated quantitatively using two different metrics that compare the quality of the final registered and averaged volume to the single reference volume.

Mean squared error (MSE) is widely recognized for its effectiveness in quantifying the intensity difference between the registered image and the reference image. It provides a clear and straightforward measure of registration accuracy in terms of pixel intensity values, which is crucial for evaluating the performance of registration algorithms in enhancing OCT image quality. The MSE has been extensively utilized in OCT studies for this purpose, reflecting its relevance and applicability to the field.^29^^,^^44^ It is the average squared difference between averaged and reference OCT intensity images, defined as

$$\mathrm{MSE}=\sqrt{\frac{1}{N}\overset{N}{\underset{n=1}{\sum }}(I_{\mathrm{avg}}-I_{\mathrm{ref}}{)}^{2}},$$ (3)

where $\sum_{n=1}^{N}$ denotes summation over the total number of pixels (N), and $I_{\mathrm{avg}}$ and $I_{\mathrm{ref}}$ represent the pixel intensity of the averaged and reference images, respectively.^29^

Structural similarity index measure (SSIM) evaluates the perceptual change in structural information, which is vital for assessing the quality of OCT images, where preserving structural details is of great importance. It can capture relevant structural changes and has been employed in various OCT image processing studies.^45^^,^^46^ Here it measures the perceptual difference between the registered image and the reference image, defined as

$$\mathrm{SSIM}(x,y)=\frac{\left(2\mu_{\mathrm{ref}}\mu_{\mathrm{avg}}+c_{1})(2\sigma_{\mathrm{ref},\mathrm{avg}}+c_{2}\right)}{\left(\mu_{\mathrm{ref}}^{2}+\mu_{\mathrm{avg}}^{2}+c_{1})(\sigma_{\mathrm{ref}}^{2}+\sigma_{\mathrm{avg}}^{2}+c_{2}\right)},$$ (4)

where ref and avg represent the *en face* mean projection of the reference and the averaged volumes, respectively. $\mu_{\mathrm{ref}}$, $\mu_{\mathrm{avg}}$, $\sigma_{\mathrm{ref},\mathrm{avg}}^{2}$, and $\sigma_{\mathrm{ref},\mathrm{avg}}$ are the average, variance, and covariance of the pixel intensity values, respectively. The terms $c_{1}$ and $c_{2}$ are two small constants added to avoid instability when $\mu_{\mathrm{ref}}^{2}+\mu_{\mathrm{avg}}^{2}$ and $\sigma_{\mathrm{ref}}^{2}+\sigma_{\mathrm{avg}}^{2}$ are equal to zero, where $c_{1}$ and $c_{2}\ll 1$.^32^

## 3 Results

### 3.1 3D Registration and Averaging of OCT/OCTA Volumes Acquired from DS-OCT System

**Fig. 6:**
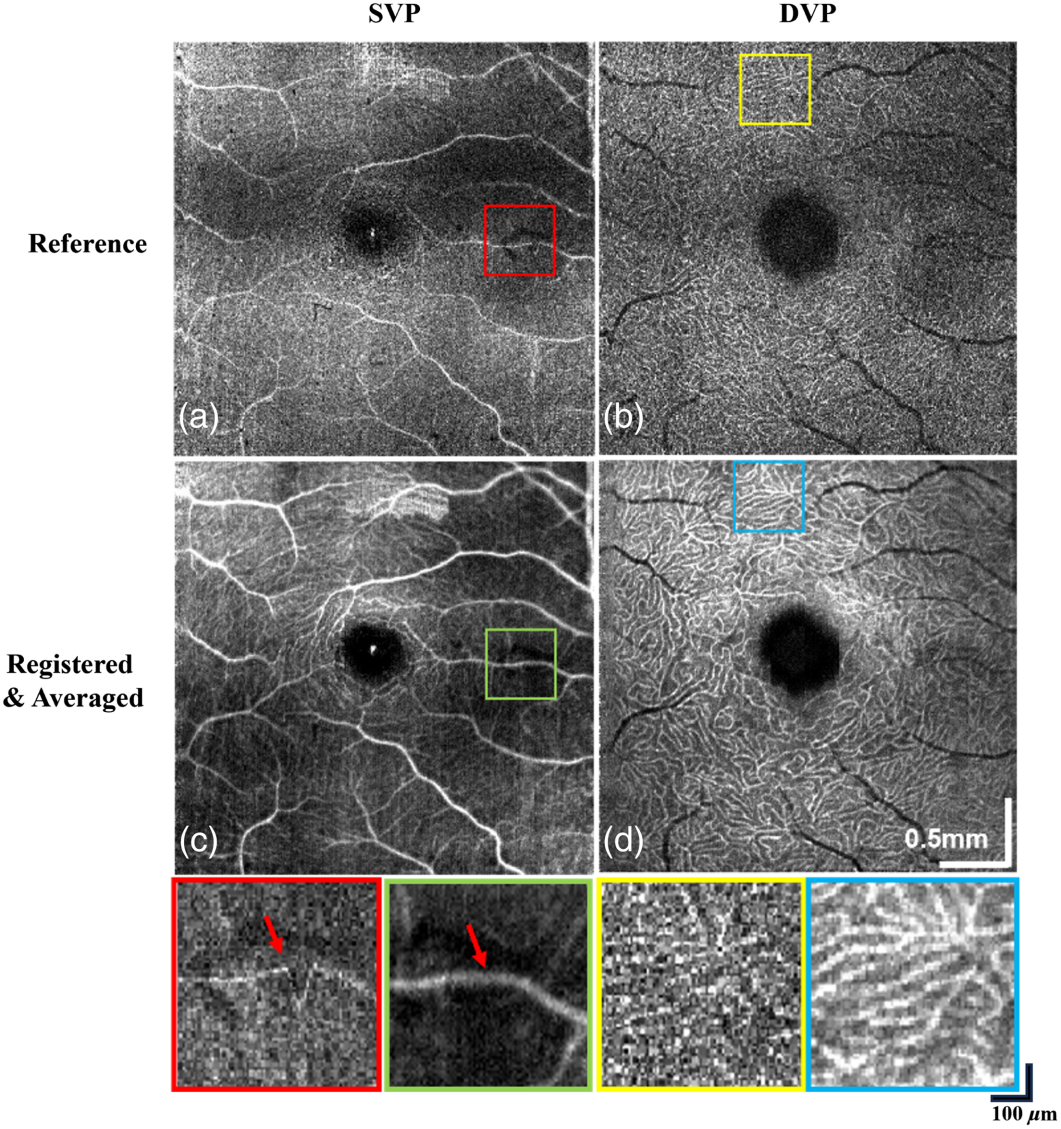
Performance of the 3D registration algorithm on custom-built DS-OCT system using high-speed mode. (a), (b) Single reference OCT volumes of SVP and DVP. The red square highlights a motion artifact, whereas the yellow square shows the disconnectivity of the deep vessels. (c), (d) 47 registered and averaged OCT volumes for SVP and DVP. Motion artifacts have been corrected as shown by the green square. Contrast improvement in the vasculature is shown by the blue square.

Using the high-speed mode of the DS-OCT system, a total of 47 OCT volumes were acquired from a healthy control eye at a 500-kHz A-scan rate, with a trade-off in SNR.^37^ The comparison between the reference and averaged volumes after the registration process is shown in Fig. 6.

The superficial vascular plexus (SVP) and deep vascular plexus (DVP) were segmented by OCTexplorer 3.8.0 (The Iowa Reference Algorithms, Retinal Image Analysis Lab, Iowa Institute for Biomedical Imaging, Iowa City, Iowa),^47^^–^^51^ followed by manual corrections to the lines that were segmented incorrectly.^27^^,^^31^^,^^32^ Figure 6(a) shows an artifact in a single volume that appears as an abrupt change in the vasculature architecture, which was corrected after volumes have been registered and averaged [Fig. 6(c)]. Furthermore, there was an improvement in the overall contrast of the image, which allows more detailed and connected vasculatures to be visualized in both vascular layers. Portions of the images enclosed in the colored squares are magnified at the bottom of each figure to display the differences between the reference and the averaged registered volumes.

**Fig. 7:**
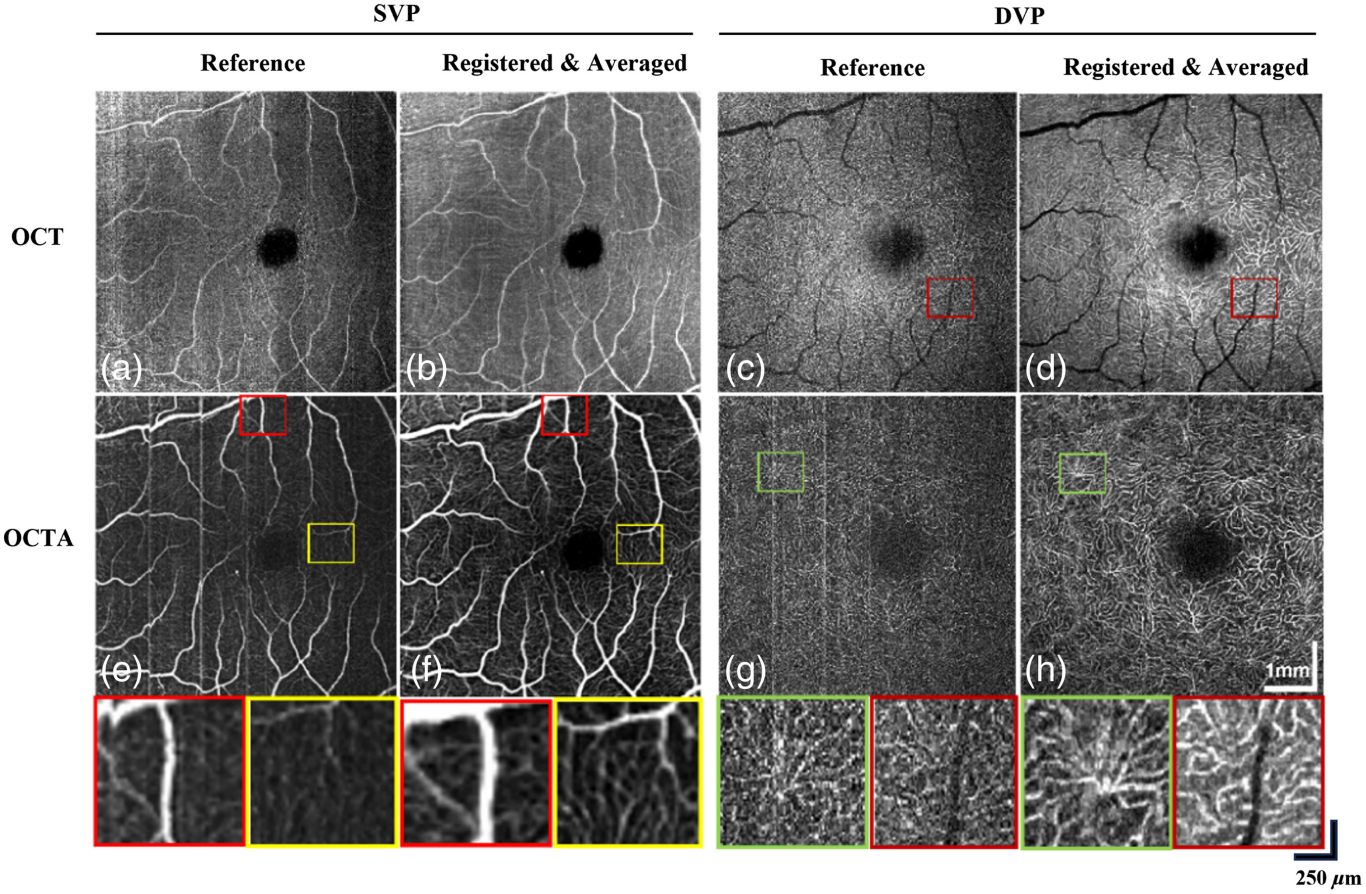
Performance of the 3D registration algorithm on custom-built DS-OCT system using high SNR mode. (a), (b) Comparison between single reference and 20 registered OCT volumes for SVP. (c), (d) Comparison between a single reference and 20 registered OCT volumes for DVP. Brighter vasculature and reduced speckle noise is shown by the red squares. (e), (f) Comparison between single reference and 20 registered OCTA volumes for SVP. Motion artifacts have been corrected in F as shown by the red squares. Contrast improvement in the vasculature is shown by the yellow squares. (g), (h) Comparison between single reference and 20 registered OCTA volumes for DVP. Motion artifacts marked by the green square have been corrected in H.

A total of 20 OCT/OCTA volumes were acquired from the same subject by the custom-built DS-OCT system using the high-SNR mode. The high-SNR mode adopts dual-balanced detection and acquires at a 250-kHz A-scan rate. Figure 7 shows the comparison between different retinal layers extracted from the OCT and OCTA volumes before and after applying the 3D registration algorithm. Despite selecting the reference volume based on the criteria described in Sec. 2.1.1, motion artifacts that appear as disconnected vessels are still present [Figs. 7(a) and 7(c)] and have been corrected after registration and averaging [Figs. 7(b) and 7(d)]. In OCTA volumes, motion artifacts caused by microsaccades are present in the form of vertical bright lines [Figs. 7(e) and 7(g)]. Magnified views of improved vessel continuity and vascular contrast show successful correction with the proposed registration algorithm as shown in Figs. 7(f) and 7(h). High-contrast microvasculature visualization is demonstrated with significant speckle noise reduction compared to the reference [Fig. 7d)].

### 3.2 3D Registration and Averaging of OCT Volumes Acquired from SAO-OCT System

**Fig. 8:**
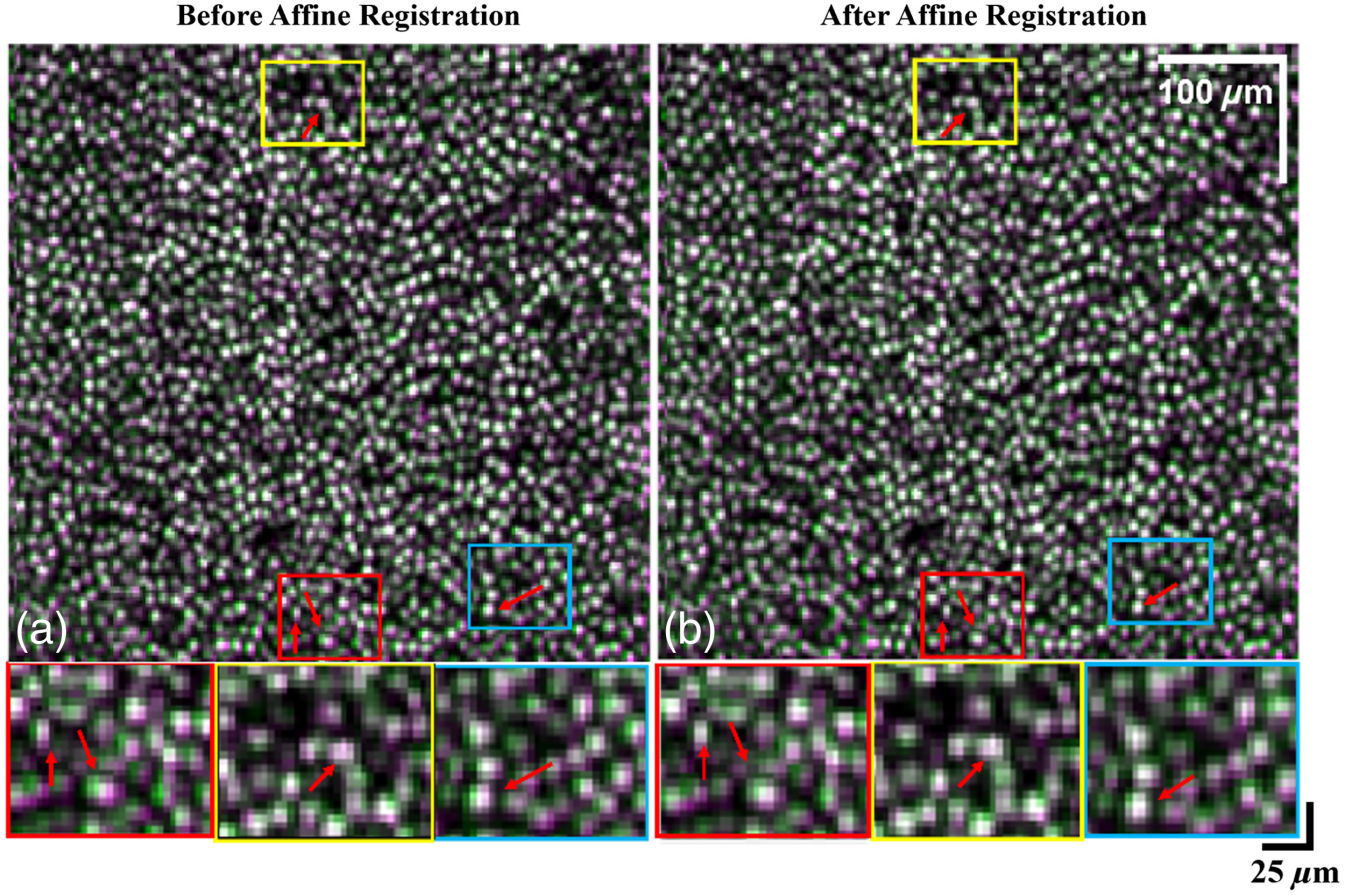
Performance of the affine registration on custom-built SAO-OCT system. (a) Cone mosaic layer overlay between reference and B-scan registered volumes prior to affine registration. (b) Cone mosaic layer overlay between reference and registered volumes after affine registration. Green and magenta pixels correspond to the reference and registered volumes, respectively. White pixels correspond to overlapping areas between the two volumes, with increased white pixels indicating a better alignment of volumes after affine registration.

A total of 88 OCT/OCTA volumes were acquired from a healthy control eye by the custom-built SAO-OCT system with image-based wavefront optimization for *in vivo* cellular imaging, focused on the fovea. To find a more precise rotational angle in affine registration, images were first upsampled by a factor of 2 and downsampled after the rotation. No artifacts appeared in the images after alignment with this technique. To validate the performance of affine registration, we compared the registered volumes with and without the affine rotation step as described in Sec. 2.2.2. Figure 8(a) presents the reference cone mosaic (green pixels) overlayed with the registered volume (magenta pixels) prior to affine registration, where the white pixels denote areas that are identical in both images. Red arrows indicate areas that are better aligned with the reference after affine registration, indicated by greater overlap between the two volumes [Fig. 8(b)].

**Fig. 9:**
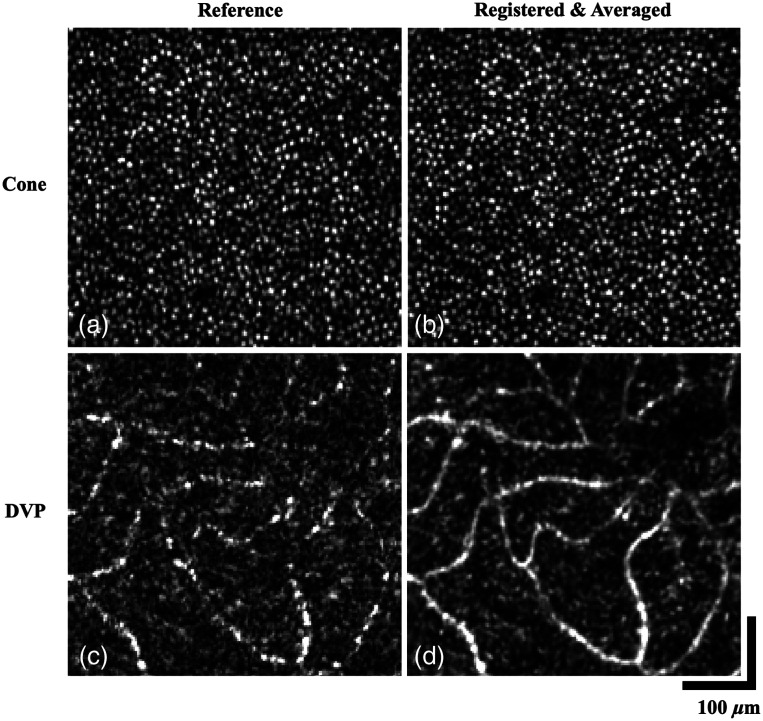
Performance of the 3D registration algorithm on custom-built SAO-OCT system. (a), (b) Cone mosaic layer of reference and registered volume. (c), (d) *En face* projection of the DVP of the reference and registered volume. Contrast improvement, vasculature connectivity, and reduction of speckle noise are noticeable.

Figure 9 presents a comparison of *en face* images depicting the cone mosaic and DVP before and after the implementation of the 3D registration algorithm. Irregularity of the cone mosaic can be seen from distortions resulting from tremors, which have been improved after registration and averaging to form a more well-defined circular pattern [Figs. 9(a) and 9(b)]. The capillary structure in DVP is not clearly resolved in the reference image [Fig. 9(c)], with degradation of vasculature contrast and vessel discontinuity due to the presence of background noise. Through multivolume registration and averaging, the visualization of the DVP is improved through increased contrast along with improved vascular connectivity and unambiguous capillary network distribution as shown in Fig. 9(d).

### 3.3 3D Registration and Averaging of OCT Volumes Acquired from Commercial OCT System

A total of 10 OCT/OCTA volumes were acquired sequentially from a patient diagnosed with central retinal vein occlusion (CRVO) using the commercialized PLEX Elite 9000 OCT system with real-time motion tracking to detect and autocompensate for eye movements. Although less displacement artifacts are present in the OCTA—*en face* images due to real-time motion tracking, the commercialized system has lower axial and lateral resolution compared to the custom-built systems, which degrades the vasculature contrast and image quality. As discussed in Sec. 2.1.2, an additional nonrigid registration step is required to compensate for the randomized coordinates generated by the motion-tracking system.

**Fig. 10:**
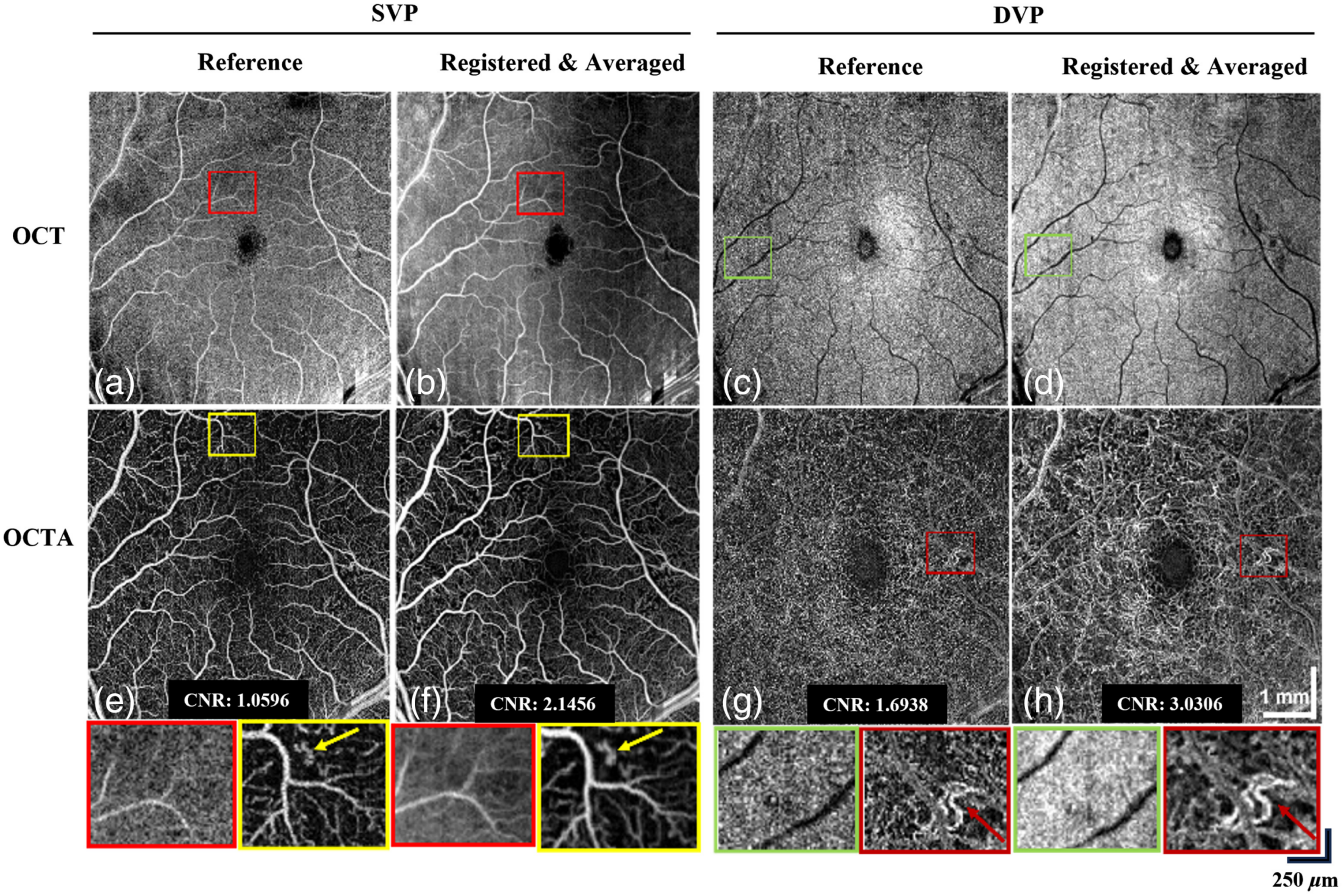
Performance of the 3D registration algorithm on commercialized OCT system (Zeiss PLEX Elite 9000) with CNR values shown for images E-H. (a), (b) Comparison between single reference and 10 registered OCT volumes for SVP. (c), (d) Comparison between a single reference and 10 registered OCT volumes for DVP. Contrast improvement in the vasculature is shown by the green squares. (e), (f) Comparison between a single reference and 10 registered OCTA volumes for SVP. Reduced speckle noise is shown by the red squares. (g), (h) Comparison between single reference and 10 registered OCTA volumes for DVP.

Figure 10(a) shows the *en face* OCT image of SVP extracted from the reference OCT volume, with disconnected vasculatures marked by the red box. 3D registration and averaging help reduce the speckle noise and increase the contrast of the blood vessels [Fig. 10(b)]. Similarly, the magnified region in Figs. 10(c) and 10(d) demonstrates the performance of 3D registration in removing background noise and improving vasculature contrast for the DVP layer. Patients diagnosed with CRVO tend to have suspended scattering particles in motion caused by hyperreflective fluid^52^ as shown by the yellow box in Fig. 10(e), which can be better visualized postregistration [Fig. 10(f)]. Figures 10(g) and 10(h) show the improved detection of neovascularization after 3D registration and averaging, with brighter and more continuous vessels. Furthermore, we have computed the contrast-to-noise ratio (CNR) of the reference and final averaged volumes using the *en face* mean projection.^32^ For SVP, there is a CNR improvement from 1.0596 in the reference volume to 2.1456 in the averaged volume. For DVP, there is a CNR improvement from 1.6938 in the reference volume to 3.0306 in the averaged volume.

### 3.4 Quantitative Analysis

**Fig. 11:**
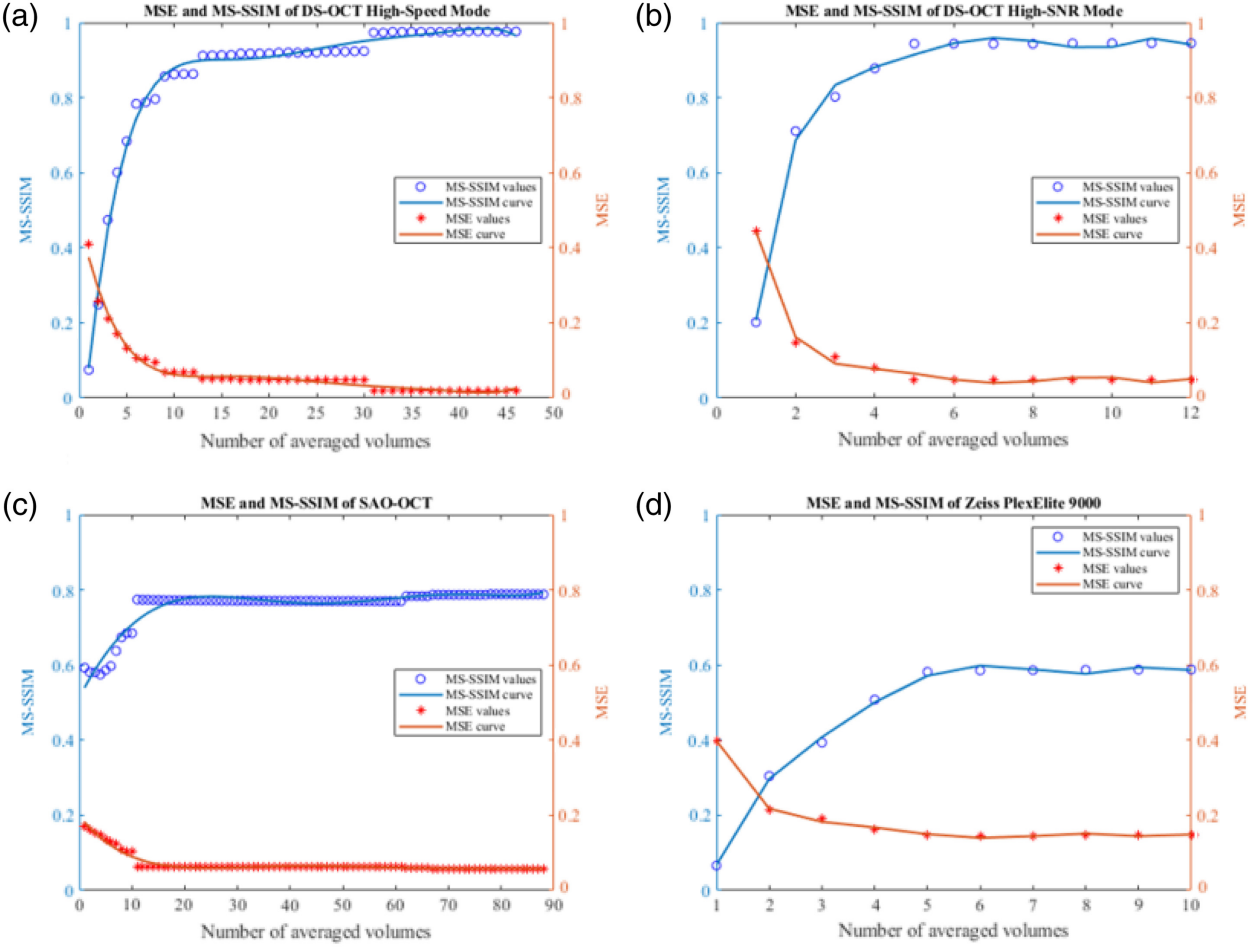
Numerical evaluation of the registration performance versus number of averaged volumes using MSE and MS-SSIM. (a) MSE and MS-SSIM evaluation of OCT volumes acquired from the DS-OCT with high-speed mode. (b) MSE and MS-SSIM evaluation of OCT volumes acquired from the DS-OCT with high-SNR mode. (c) MSE and MS-SSIM evaluation of OCT volumes acquired from the SAO-OCT system. (d) MSE and MS-SSIM evaluation metrics of OCT volumes acquired from commercialized OCT system (Zeiss PLEX Elite 9000).

To further evaluate the performance of the proposed registration algorithm, MSE and MS-SSIM were calculated for each postprocessed volume compared to the reference using the mean *en face* projection of the volumes. Figure 11 illustrates how the MSE (orange) and MS-SSIM (blue) values improve after increasing the number of averaged volumes, for different OCT systems with varying specifications. Table 2 summarizes the initial and final values of each graph, where the initial value is calculated from a single registered volume and the final value is calculated from averaging a number of registered volumes. In all cases, MSE decreases with an increase in averaged volumes, while MS-SSIM increases. Both quantification methods indicate an improvement in registration quality by measuring an increase in similarity between the reference and final averaged volumes.

**Table 2:** Initial and final values of the MS-SSIM and MSE.

System	MS-SSIM		MSE		# of volumes
	Initial value	Final value	Initial value	Final value	
DS-OCT (high-speed mode)	0.0748	0.9765	0.4090	0.0195	47
DS-OCT (high-SNR mode)	0.2019	0.9454	0.4458	0.0484	12
SAO-OCT	0.5931	0.7883	0.1705	0.0577	88
Plex Elite 9000	0.0660	0.5879	0.3989	0.1478	10

## 4 Discussion

In this study, an effective and robust 3D registration algorithm was introduced for correcting motion artifacts and improving image contrast for various OCT retinal images. The registration process was performed in three sequential steps: preprocessing, B-scan registration, and affine registration. In the preprocessing stage, the reference volume was automatically selected based on minimal motion artifacts and was used to align the remaining target volumes. Furthermore, the axial matching algorithm was used to align the volumes along the depth, followed by automatic motion artifact removal. The B-scan registration was implemented to align the position of the B-scans in the volumes laterally, which was followed by affine registration to correct for the finer details in the images, such as rotational differences. It is worth noting that our algorithm relies solely on OCT amplitude data, as we aim for broad applicability across different OCT platforms. For example, certain OCT systems, such as swept-source OCT experience phase instability, which can compromise the reliability of phase-based registration methods.^53^ In the context of our algorithm, amplitude and intensity attributes are inherently more stable and consistent across systems, ensuring that the algorithm remains effective, irrespective of the underlying phase stability of the OCT system being used. Furthermore, due to the sensitivity of the phase term, the time interval between each volume acquisition can vary slightly, and we cannot guarantee that the time intervals between consecutive volumes are necessarily short enough for phase stability in the OCT signal.

We demonstrated several benefits of the 3D registration for averaging serially acquired OCT images, and a summary of its comparison to existing OCT registration methods can be found in Table 3. In contrast to many existing algorithms that rely on OCTA vascular contrast for registration, our approach uses structural OCT data for enhanced versatility across diverse OCT systems. By leveraging structural OCT volumes that are readily available in all systems, our algorithm ensures broader applicability while still accommodating OCTA. Some of its advantages include but are not limited to: (i) detection and removal of motion artifacts arising from microsaccades along the slow scan direction using the automatic motion removal approach; (ii) better visualization of microvasculature in the retina; and (iii) applicability of our algorithm for different types of OCT systems, such as custom-built and commercially available systems. Several key distinct system parameters were presented, such as SNR, axial, and lateral resolution, to illustrate the performance of the algorithm, which has proven to be robust enough to provide reliable registration for most subjects, including patients with CRVO, as demonstrated in Sec. 3.3. In Fig. 11 and Table 2, we quantitatively assessed the performance of the proposed algorithm by computing the MSE and MS-SSIM metrics using corrected volumes obtained from different system specifications. Across all datasets, a consistent trend emerged as the number of averaged volumes increased, where MSE decreased while MS-SSIM increased. This pattern indicates a reduction in MSE and an improvement in MS-SSIM between the reference and averaged volumes, affirming the efficacy of our algorithm in registering multiple volumes to a single reference volume and validating its adaptability to various imaging specifications. Moreover, the observed trends strongly suggest that increasing the number of averaged volumes leads to enhanced final image quality.

**Table 3:** Summary of previous OCT registration methods compared to the proposed method.

Reference	Method	Modality	λ (nm)	FOV (mm×mm)	Resolution AR (μm) × LR (μm)
Cheng et al.^27^	Affine and B-spline transformation	SS-OCT	1310	9×9	11.1×11.1
		Zeiss PLEX Elite 9000	1060	6×6	6.3×20.0
Athwal et al.^28^	CC, SIFT, NCC	SS-OCTA	1060	2×2	6.3×7.7
Kurokawa et al.^29^	POC, NCC	AO-OCT	790	1×1	5.3×2.9
Heisler et al.^32^	CC, SIFT, NCC	SS-OCTA	1060	2×2	6.0×8.6
Zang et al.^54^	Cost-function optimization	SS-OCTA	1045	6×10	5.6×8.9
Proposed method	POC, CC	DS-OCT	810	6×6	3.2×9.7
		SAO-OCT	1060	0.5×0.5	7.0×3.3
		Zeiss PLEX Elite 9000	1060	6×6	6.3×20.0

SS, swept-source; CC, cross correlation; SIFT, scale-invariant feature transform; NCC, normalized cross correlation; POC, phase-only correlation; AO, adaptive-optics; AR, axial resolution; and LR, lateral resolution.

For SAO-OCT discussed in Sec. 3.2, we observed a notable difference in improvement between the cone mosaic images and the blood vessels at the DVP in Fig. 9. This discrepancy can be attributed to the distinct characteristics of these structures. The DVP comprises moving particles that randomize the scattering of light, appearing disconnected or less defined in a single volume compared to the averaged volume, where multivolume averaging aids in enhancing the clarity of vascular structures. Conversely, photoreceptors are static cellular structures, and the alterations in their appearance after 3D registration and averaging are not as prominent as those observed in the DVP. Additionally, it is worth noting that wavefront correction is focused on the photoreceptor layer during acquisition, which further contributes to the higher single volume image quality in this region.

Despite the ability of our algorithm to enhance retinal plexus visualization by registering and averaging multiple sequentially acquired OCT volumes, we acknowledge several limitations of our study. First, we assumed that the acquisition time of each fast B-scan is short enough to neglect head motion. We also assume that the acquisition time for a single fast B-scan is short enough to avoid microsaccades within the fast B-scan. For the OCT systems used in this study, the systems acquire at a minimum of 100 KHz A-scan rate, deeming this assumption possible as microsaccades typically have an average duration in the order of milliseconds.^16^^,^^55^ Second, the FOV of the final volume after registration and averaging is limited by the FOV of the reference volume. To address this limitation, multiple reference volumes can be selected by ranking the volumes from high to low quality, and results obtained from each reference can be combined to create a volume with a larger FOV. Third, projection artifacts associated with superficial vessels may be present in the DVP images and final registered volumes, which may obscure deep vessel structures even after accurate segmentation. These projection artifacts can be identified by comparing the vascular structures in both SVP and DVP for similar patterns. Future studies should consider the use of 3D projection artifact compensation methods, such as projection-resolved OCTA,^56^ in order to improve the 3D visualization of retinal microvasculature. Additionally, it is important to acknowledge that our algorithm has been tested on only one pathological case. We aim to expand our testing to encompass a broader range of patients with various retinal pathologies to further validate its efficacy across different clinical scenarios. Furthermore, it is worth noting that while the lowest acquisition speed demonstrated in this manuscript is 100 kHz—the Zeiss PlexElite 9000—future work will focus on extending our evaluation to a wider range of systems, including lower-speed systems, such as the Heidelberg Spectralis (Heidelberg Engineering Gmbh, Germany) which operates at 85 kHz, as well as higher-speed systems operating in the MHz range, such as OCT systems with Fourier domain mode locking lasers.^57^ Finally, since there is no gold standard to evaluate the performance of the registration algorithm and verify optimal alignment in the final image, current evaluation methods can only assess contrast and SNR improvement, as well as the similarity between images using statistical measurements. Despite a lack of gold standard metrics to compare registration methods due to highly specialized datasets and system configurations in OCT, we have confirmed the performance of the proposed algorithm with control eyes, demonstrating its reliability across various OCT systems.

## 5 Conclusion

OCT is a powerful imaging technique widely used in ophthalmology for studying various ocular diseases due to its noninvasive property and ability to provide high-resolution, depth-resolved images for diagnosis, and disease monitoring. However, a critical challenge in OCT imaging lies in patient-induced motion artifacts that can severely degrade the image quality in a single volume. Our proposed algorithm has addressed this issue by iterating upon previous works to develop a 3D registration algorithm, combining B-scan and affine registrations to correct translational shifts laterally and axially, and rotational angle differences between OCT volumes acquired sequentially. Importantly, the method is versatile and applicable to various OCT systems with different system parameters. The validation of the algorithm’s performance involved acquiring retinal images from different OCT modalities, including both custom-built and commercially available systems. The results demonstrate the algorithm’s effectiveness in reducing motion artifacts, improving image contrast, and enhancing the visualization of retinal microvasculature, which are crucial elements for the precise diagnosis and monitoring of retinal diseases.

## Data Availability

The datasets generated and analyzed during the current study are available from the corresponding author upon request.
